# Age-integrated artificial intelligence framework for sleep stage classification and obstructive sleep apnea screening

**DOI:** 10.3389/fnins.2023.1059186

**Published:** 2023-06-14

**Authors:** Chaewon Kang, Sora An, Hyeon Jin Kim, Maithreyee Devi, Aram Cho, Sungeun Hwang, Hyang Woon Lee

**Affiliations:** ^1^Computational Medicine, System Health Science and Engineering Program, Ewha Womans University, Seoul, Republic of Korea; ^2^Department of Communication Disorders, Ewha Womans University, Seoul, Republic of Korea; ^3^Department of Neurology, Korea University Ansan Hospital, Ansan, Republic of Korea; ^4^Department of Neurology, Ewha Womans University School of Medicine, Seoul, Republic of Korea; ^5^Department of Nursing Science, Ewha Womans University, Seoul, Republic of Korea; ^6^Department of Neurology, Ewha Womans University Mogdong Hospital, Seoul, Republic of Korea; ^7^Department of Medical Science, Ewha Womans University School of Medicine and Ewha Medical Research Institute, Seoul, Republic of Korea

**Keywords:** polysomnography, electroencephalography, sleep staging, obstructive sleep apnea, age-integrated, machine learning, artificial intelligence

## Abstract

**Introduction:**

Sleep is an essential function to sustain a healthy life, and sleep dysfunction can cause various physical and mental issues. In particular, obstructive sleep apnea (OSA) is one of the most common sleep disorders and, if not treated in a timely manner, OSA can lead to critical problems such as hypertension or heart disease.

**Methods:**

The first crucial step in evaluating individuals’ quality of sleep and diagnosing sleep disorders is to classify sleep stages using polysomnographic (PSG) data including electroencephalography (EEG). To date, such sleep stage scoring has been mainly performed manually *via* visual inspection by experts, which is not only a time-consuming and laborious process but also may yield subjective results. Therefore, we have developed a computational framework that enables automatic sleep stage classification utilizing the power spectral density (PSD) features of sleep EEG based on three different learning algorithms: support vector machine, k-nearest neighbors, and multilayer perceptron (MLP). In particular, we propose an integrated artificial intelligence (AI) framework to further inform the risk of OSA based on the characteristics in automatically scored sleep stages. Given the previous finding that the characteristics of sleep EEG differ by age group, we employed a strategy of training age-specific models (younger and older groups) and a general model and comparing their performance.

**Results:**

The performance of the younger age-specific group model was similar to that of the general model (and even higher than the general model at certain stages), but the performance of the older age-specific group model was rather low, suggesting that bias in individual variables, such as age bias, should be considered during model training. Our integrated model yielded an accuracy of 73% in sleep stage classification and 73% in OSA screening when MLP algorithm was applied, which indicates that patients with OSA could be screened with the corresponding accuracy level only with sleep EEG without respiration-related measures.

**Discussion:**

The current outcomes demonstrate the feasibility of AI-based computational studies that when combined with advances in wearable devices and relevant technologies could contribute to personalized medicine by not only assessing an individuals’ sleep status conveniently at home but also by alerting them to the risk of sleep disorders and enabling early intervention.

## 1. Introduction

Sleep is an essential part in human life. Poor sleep quality can lead to reduced physical performance and have a negative impact on cognitive functions (Yuan et al., 2019). The number of patients with sleep disorders has been constantly increasing due to light pollution at night, shift work, and altered lifestyles with the recent pandemic of COVID-19 (Marvaldi et al., 2021). One of the most common sleep disorders is obstructive sleep apnea (OSA), which is estimated to affect one-seventh of the global population (Lyons et al., 2020). Patients with OSA have interrupted sleep because they repeatedly stop and resume breathing while they are asleep. They not only have difficulty with daytime activities but can also develop serious health concerns, such as hypertension and heart problems, if their OSA is not treated in a timely manner (Kumari et al., 2020).

To evaluate sleep quality and diagnose sleep disorders, polysomnography (PSG) is widely used. PSG measures various bio-signals including electroencephalography (EEG), electromyography (EMG), and electrooculography (EOG) signals and respiratory and cardiac activities. Through comprehensive analyses using the acquired multimodal data, individuals’ sleep conditions are assessed. A crucial first task in such analyses is to classify sleep stages based on the PSG data (mainly, sleep EEG). To date, sleep scoring has been performed manually by experts following standardized manuals such as the American Academy of Sleep Medicine (AASM) manual (Loh et al., 2020). According to the AASM manual, sleep is classified into the following five stages: wake, rapid eye movement (REM), and three non-REM (NREM) stages including N1, N2, and N3. Experts divide the acquired PSG data into 30-s epochs and then assign sleep stages to each epoch according to standardized criteria. Sleep stage scoring can be a time-consuming, laborious process because it is performed manually and because the time length of one full night of collected PSG data is approximately 7–8 h. Another problem with traditional sleep scoring is that the results might be subjective depending on which experts conducted the scoring. Therefore, it is desirable to devise an automatic process for sleep staging to overcome the abovementioned difficulties.

OSA is diagnosed by comprehensive analyses using questionnaires and various bio-signals obtained during PSG. In particular, airflow *via* a nasal pressure sensor and thermistor, oxygen saturation *via* pulse oximetry, and respiratory effort *via* chest and abdominal belts are used as important indices. EEG, electrocardiography (ECG), EOG, and EMG signals emitted during sleep are also used for diagnosis (Gottlieb and Punjabi, 2020). Based on these bio-signals, indices to evaluate sleep quality, including total sleep time, time spent in each sleep stage, frequency of arousal, and apnea–hypopnea index (AHI, the number of apnea and hypopnea events per hour of sleep) are calculated, which determine the presence and severity of OSA (Patil et al., 2007). Recent studies have demonstrated that there are significant differences in sleep EEG between OSA and healthy groups (Kumari et al., 2020; Kang et al., 2021), suggesting the need to focus more on sleep EEG. In particular, Kang et al. (2021) demonstrated marked differences in the power spectral densities (PSDs) of beta and sigma frequency bands and indicated that those differences were more pronounced during NREM than during REM stages.

Meanwhile, following the development of artificial intelligence (AI) technologies, recently, the studies attempting to automatically classify the sleep stages based on PSG data are increasing (Zhao et al., 2019; Sekkal et al., 2022). In particular, studies that have built sleep stage scoring models by applying conventional machine learning techniques, such as linear discriminant analysis (Liang et al., 2012; Long et al., 2013), k-nearest neighbors (kNN) (Li et al., 2012), and support vector machine (SVM) (Willemen et al., 2013; Huang et al., 2014; Wu et al., 2014; Zhu et al., 2014; Acharya et al., 2015; Enshaeifar et al., 2015), and overall, they have shown a classification performance of 70–90%. More recently, studies utilizing deep learning and artificial neural networks have been reported, where they adopted various architectures, including convolutional neural networks (CNNs; Chambon et al., 2018; Mikkelsen and De Vos, 2018; Supratak and Guo, 2020), recurrent neural networks (RNNs; Malafeev et al., 2018; Phan et al., 2018, 2019), deep neural networks (DNNs; Wei et al., 2018), or combinations thereof, such as CNN + RNN (Biswal et al., 2018; Stephansen et al., 2018; Korkalainen et al., 2019; Mousavi et al., 2019) and DNN + RNN (Dong et al., 2018). These state-of-the-art studies have further improved the performance of sleep stage classifications to the 80–90% level, without explicitly defining classification rules or features of each sleep stage. However, depending on the complexity of the model, more computational resources and time are required, thus, the trade-off between model accuracy and computational load may need to be considered, depending on the circumstances (e.g., in a mobile device-dependent environment) (Janiesch et al., 2021).

To date, studies in this area have been mainly conducted based on data obtained from healthy subjects (particularly younger adults). Recently, machine learning studies using large datasets including data from patients with sleep disorders and subjects with wide age ranges have been reported (Korkalainen et al., 2019; Jarchi et al., 2020; Sharma et al., 2021; Hussain et al., 2022). In particular, Korkalainen et al. (2019) proposed a deep learning-based sleep stage classification model using a clinical dataset of patients with suspected OSA and demonstrated that classification accuracy decreased as the OSA severity increased (84.5% for individuals without OSA; 76.5% for severe OSA patients). Sharma et al. built the models by applying six traditional machine learning classifiers (decision trees, logistic regression, naive Bayes, SVM, kNN, and ensemble bagged trees) based on a dataset, which included healthy subjects and patients with multiple sleep disorders, including insomnia, narcolepsy, REM behavior disorder, etc., and achieved a maximum accuracy of 85% (Sharma et al., 2021). However, studies based on large clinical datasets are still limited, while few studies have systematically investigated the effects of individual variables such as the presence of sleep disorders, age, and gender in subjects.

Previous studies with sleep EEG analysis have revealed that even healthy individuals without sleep disorders may have different characteristics depending on their age. It has been found that not only does the time consumed for slow-wave sleep (SWS) decreases with aging but also the power of the activity itself during SWS is reduced (Landolt et al., 1996; Campos-Beltrán and Marshall, 2021). In addition, the sleep spindle, which is the key feature of the N2 stage (Werth et al., 1997; Fogel and Smith, 2011), has been found to decrease in its amplitude, density, and length with aging (Campos-Beltrán and Marshall, 2021). Therefore, in building a model that automatically evaluates sleep EEG, it is necessary to consider the age of subjects in the training dataset and to systematically analyze the effect thereof.

In this study, we first built a machine learning model that automatically performs sleep stage classification using the PSD features of sleep EEG and three different algorithms: SVM, kNN, and multilayer perceptron (MLP). In particular, we analyzed the age-related effects by constructing a general model trained on the data of all subjects regardless of their age and age-specific models (younger and older group models) and compared their performance. We then conducted OSA screening based on a model trained on EEG features for each sleep stage and evaluated its feasibility. Therefore, we provide a comprehensive computational framework that automatically scores sleep stages and further determines the risk of OSA.

## 2. Methods

### 2.1. Data acquisition and preprocessing

Data from a total of 139 subjects from standard PSG conducted at Ewha Womans University Mokdong Hospital were employed in this study (Table 1). The age of the subjects ranged from 18 to 65 years. To investigate age-related effects, we divided the subjects into two groups: a younger group aged 18–45 years, including young and early middle-aged adults, and an older group aged 46–65 years, including late middle-aged adults (Medley, 1980). The number of subjects in the younger and older groups were 69 and 70, respectively. The dataset included 42 healthy subjects and 97 patients with OSA. The patients with OSA were further subdivided into mild to moderate (mtom) and severe groups according to clinical indices evaluating the severity (Gul et al., 2018), and the values in each group for Respiratory Disturbance Index (RDI) and blood oxygen saturation (SpO_2_), which are key clinical indices, are presented in Table 1. To examine only the effects of OSA, all subjects with other medical histories such as stroke, neurological disorders, alcoholism, cancer, hypertension, and thyroid problems were excluded. More detailed demographic characteristics for the 139 subjects are provided in Table 1.

**Table 1:** Demographic characteristics of the subjects involved in this study.

	Younger group (aged 18–45 years)	Older group (aged 46–65 years)
Numbers of subjects	69	70
Age (mean ± SD)	32.91 ± 8.9	52.47 ± 7.22
OSA diagnosis (N, RDI, SpO_2_)		
Healthy	22 (RDI = 4.35 ± 15.33, SpO_2_ = 89.40 ±8.07)	20 (RDI = 5.97 ± 10.03, SpO_2_ = 88.4 ± 6.41)
Mild to moderate OSA	25 (RDI = 15.90 ± 9.07, SpO_2_ = 82.92 ± 4.39)	24 (RDI = 12.46 ± 11.12, SpO_2_ = 83.70 ± 5.84)
Severe OSA	22 (RDI = 53.51 ± 26.61, SpO_2_ = 73.40 ± 10.95)	26 (RDI = 51.05 ± 20.15, SpO_2_ = 74.92 ± 7.90)
Gender (N)		
Female	8	24
Male	61	46
Body mass index (mean ± SD)	27.12 ± 5.23	26.39 ± 3.95
Smoking status (N)		
Never	42	42
Ex-smoker	7	15
Current	20	13
Number of epochs for each sleep stage		
(total, mean ± SD)		
Wake	8,027 (116.33 ± 103.16), 14.24%	8,218 (117.40 ± 72.13), 14.63%
N1	12,234 (117.30 ± 118.89), 21.71%	13,365 (190.93 ± 117.64), 20.23%
N2	25,799 (373.90 ± 122.47), 45.78%	25,167 (359.52 ± 114.79), 44.81%
N3	2,502 (36.26 ± 47.24), 4.44%	1,181 (16.87 ± 30.70), 2.10%
REM	7,796 (112.90 ± 50.90), 13.83%	8,235 (117.64 ± 51.73), 14.66%

Data from all subjects were collected with Twin PSG Clinical Software (Glass Technologies, Warwick, RI, United States) (Choi et al., 2021). Full night sleeps of approximately 5–7 h were recorded for each subject. Sleep EEG was recorded using six electrodes (F3, F4, C3, C3, O1, and O2) following the international 10–20 system. A single ground electrode was attached on the forehead, and two linked ear electrodes were used as references. Impedances of electrodes were kept under 10 kΩ and the sampling rate was 200 Hz. A more detailed description of PSG data acquisition can be found in our previous paper (Choi et al., 2021). The sleep EEG was segmented into 30-s epochs, and each epoch was scored as one of five sleep stages (wake, N1, N2, N3, REM), as inspected by experts following the AASM manual (version 2.6; Berry et al., 2020).

The artifacts of EEG tend to contribute to the features of distinct sleep stages (Brunner et al., 1996); therefore, preprocessing of the sleep EEG was performed simply with bandpass filtering of 0.5–50 Hz. Out of data from 139 subjects, data from 111 subjects were used for the training set, and the remaining data (from 28 subjects) were used for the test set. The training and test sets were evenly distributed by age and OSA diagnosis.

### 2.2. Integrated AI framework for sleep staging and OSA screening

**Figure 1:**
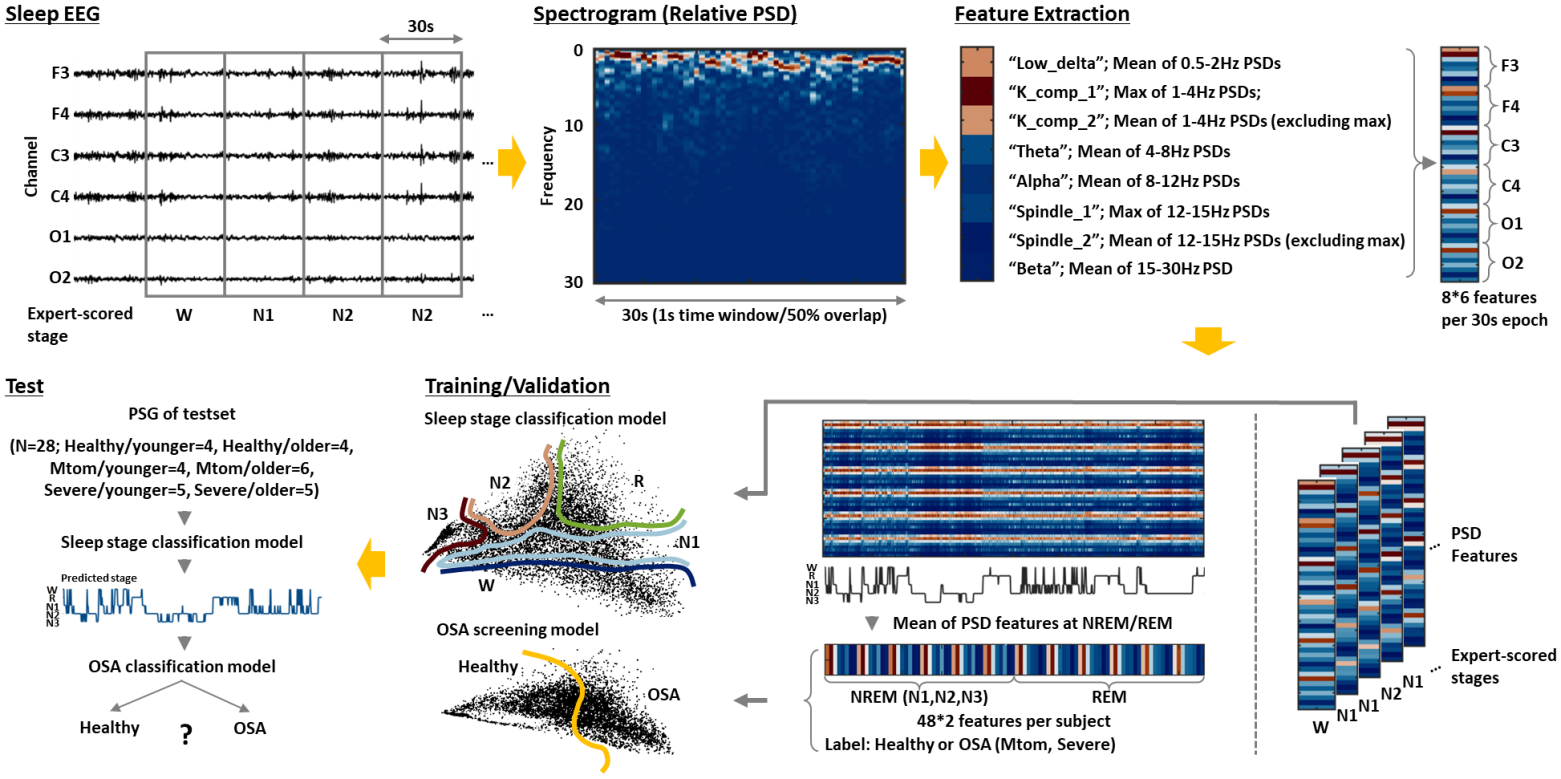
Integrated AI framework for sleep staging and OSA screening. The sleep EEG acquired from each subject is divided into 30-s epochs after preprocessing. Then, a spectrogram of the epoched signal is derived through short-time Fourier transform. Based on this, the following eight features are extracted: low_delta, K_comp_1, K_comp_2, theta, alpha, spindle_1, spindle_2, and beta. The feature extraction is performed on signals obtained from each channel (F3, F4, C3, C4, O1, and O2); thus, a total of 48 features are derived per epoch. A sleep stage classification model is constructed by training those features and the sleep stage labels scored by experts using the three different algorithms: SVM, kNN, and MLP. An OSA screening model is further built by training the average features of the REM and NREM (N1, N2, and N3) stages and OSA diagnosis for each subject. Based on this integrated model, as sleep EEG data from new subjects are input, the model can automatically analyze them to classify sleep stages and inform the risk of OSA for each individual.

We propose an integrated AI framework that automatically classifies sleep stages by analyzing the acquired sleep EEG and further screens OSA based on those results. The overall pipeline on this is depicted in Figure 1. The preprocessed sleep EEG was divided into 30-s epochs in the same manner as experts review, and eight main features were extracted from the spectrogram obtained *via* time-frequency analysis of the signal at each epoch. The features were extracted from the signals for each channel. A more detailed description for feature extraction is provided in the following section. A sleep stage classification model was built by training the features for each epoch and the labels (sleep stage) scored by experts based on three different algorithms: SVM, kNN, and MLP. An OSA screening model was built by training the eight features in the REM and NREM (N1, N2, N3) stages of the healthy and OSA groups using the corresponding algorithms. In particular, to examine the age-related effect, a general model trained on the data of all subjects regardless of their age and age-specific models trained on only data from each age group were separately constructed, and their performance was compared. Each model was verified through five-fold cross-validation repeated 10 times, and the performance of the final model was further evaluated using the test set that was uniformly extracted according to the age and OSA diagnosis.

### 2.3. Feature extraction

For model training for sleep stage classification, we employed frequency-domain features that can directly quantify specific patterns crucial for discriminating sleep stages with relatively simple computations (Aboalayon et al., 2016; Malafeev et al., 2018). According to the AASM manual, the epoch is labeled ‘wake’ if 50% or more of the signal has an alpha rhythm. Stage N1 is scored when the alpha rhythm is attenuated and replaced with lower amplitude, mainly theta power. Stage N2 is similar to N1, except that the epoch has unique features called a K complex and sleep spindle (Krauss et al., 2018; Ioannides et al., 2019). The K complex refers to a large abrupt activity with a delta frequency component, and the sleep spindle refers to a brief burst of the sigma band. They occur approximately every two epochs and have a duration of 0.5–1.5 s (Huang et al., 2014). Finally, stage N3 is scored when the slow waves of delta band continue (particularly low delta components of 0–2 Hz (Huang et al., 2014)).

To consider the abovementioned characteristics, we first performed a short-time Fourier transform (STFT) using MATLAB (version R2019b) to derive the spectrogram of the signal at each epoch. The STFT was calculated based on time windows of 1 sec with 50% overlap, resulting in 59 PSD vectors in one epoch (30 s). PSD vectors derived from each time window were normalized by their sum. The frequency band was divided into low delta (0.5–2 Hz), delta (1–4 Hz), theta (4–8 Hz), alpha (8–12 Hz), sigma (12–15 Hz), and beta (15–30 Hz) bands, and eight features to be used for model training were extracted from these results. In particular, to detect the K complex and sleep spindle, maximum values of 59 relative PSDs were extracted in the delta and sigma bands, respectively, and the mean values of the remaining 58 values were also extracted (Huang et al., 2014). For other frequency bands, an average value of 59 relative PSDs was extracted (Figure 1). Consequently, a total of 48 features were derived per epoch because eight features were extracted from the signals of each channel (F3, F4, C3, C4, O1, and O2).

Referring to a previous paper that demonstrated significant differences in sleep EEG characteristics in REM and NREM stages between healthy and OSA groups (Kang et al., 2021), we derived the average features corresponding to the REM and NREM (including N1, N2, and N3) stages based on the extracted features for each epoch, and used those features for OSA screening model training.

### 2.4. Model training

Both the sleep stage classification and the OSA screening models were trained with a supervised machine learning approach that learns the extracted EEG features and labels assigned by experts (sleep stages or OSA diagnosis). For the model training, three different algorithms were employed including SVM, kNN, and MLP.

Regarding the kernel for the SVM, the radial basis function (RBF) was used (Huang et al., 2014). The kernel trick is remapping to a different plane or dimension to obtain a decision boundary, wherein the SVM operates with two hyperparameters called C and gamma. A grid search was performed to set the parameter values, within a set C [0.1, 1, 10, 100] and gamma [0.001, 0.01, 0.1, 1, 10], and the best parameters calculated per the experiments were applied. With respect to the hyperparameters of kNN, the number of neighbors (n_neighbors), weights between the neighbors, and metric used for distance calculation were adjusted, and the best parameter values acquired from the grid search, within a set n_neighbors [1, 3, 5, 7, 9, 11, 13, 15, 17, 19], weights [‘uniform’, ‘distance’], and metric [‘euclidean distance’, ‘manhattan distance’], were used. For the MLP, the number of hidden layers and the number of nodes for each hidden layer that determine the neural network structure were used as hyperparameters. The optimal combination was derived and used through the grid search, within a set the number of hidden layers [1, 2] and the number of nodes for each hidden layer [16, 32, 64]. Weight optimization at each node was performed based on Adam, which is a stochastic gradient-based optimization algorithm (Kingma and Ba, 2014), and a rectified linear unit activation function, and the maximum number of iterations was set to 200.

### 2.5. Model validation and performance evaluation

Five-fold cross-validation was repeated 10 times on the built models, and the performance between the models was statistically analyzed based on the 50 accuracy values derived from this assessment. Comparisons between the built models or groups were performed through one-way ANOVA, which is based on the F-statistic (Verma, 2013). ANOVA results are presented as *p*-values and *F*-values with degrees of freedom (between and within groups). The significance level was defined as *p* < 0.01, and post-hoc analyses were performed for significant results using independent-samples t-test with Bonferroni correction.

The performance of the final models was further assessed by employing the test set evenly extracted depending on age and OSA diagnosis.

## 3. Results

### 3.1. Sleep stage classification

#### 3.1.1. Performance comparison between the general model and age-specific models

**Figure 2:**
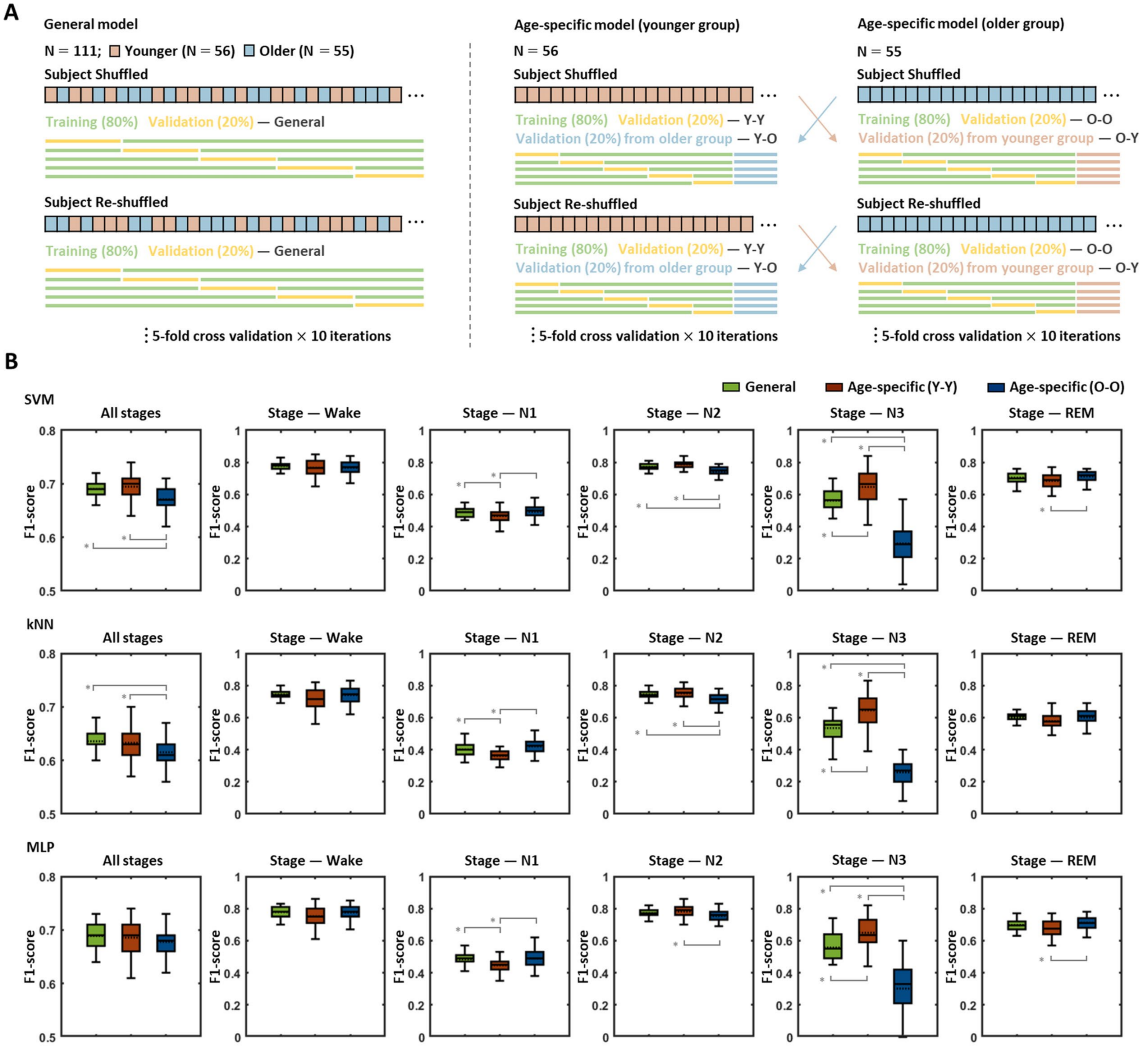
Performance comparison between the general model and age-specific models. **(A)** Model training and validation procedures. Each model was assessed by five-fold validation repeated 10 times, and every five-fold validation was conducted based on a newly shuffled dataset. The general model was trained and validated based on the data from all subjects, the age-specific models were trained with the data corresponding to each age group, and the validation was performed using data belonging to their own age group (Y-Y, O-O) as well as data from the other age group (Y-O, O-Y). **(B)** Validation results for the general model and age-specific models. The figures show the results obtained by the validation procedures of each model for each sleep stage. Pairs with statistically significant differences in classification accuracy are indicated by solid gray lines. An asterisk represents a significant difference (* *p* < 0.01).

The sleep stage classification models were built based on three different learning algorithms (SVM, kNN, and MLP), and each model was evaluated *via* five-fold cross-validation repeated 10 times, in which each iteration was performed based on a newly shuffled dataset (Figure 2A). To investigate the age-related effects, two types of models were built with each learning algorithm, and their performance was assessed: a general model trained with data from all subjects regardless of age and age-specific models trained only with data from younger or older subjects. The age-specific models were validated using subjects’ data in their own age-group (Y-Y, O-O) as well as data from the other age-group (Y-O, O-Y).

The classification accuracy for each sleep stage (wake, N1, N2, N3, REM) and the overall accuracy across all stages of the abovementioned models are presented in Figure 2B. In the SVM-based models, statistical analyses demonstrated significant differences among the accuracies of the general model and age-specific models [in all stages: *F*(2,147) = 15.27, *p* < 0.001; wake: F(2,147) = 7.35, *p* = 0.239; N1: F(2,147) = 8.88, *p* < 0.001; N2: F(2,147) = 35.99, *p* < 0.001; N3: F(2,147) = 141.92, *p* < 0.001; REM: F(2,147) = 14.64, *p* = 0.002], and group pairs with significant differences in each post-hoc analysis are indicated by gray lines in Figure 2B (top row). The overall performance of the younger group model showed no noticeable difference with that of the general model, whereas the older group model revealed significantly poorer performance than the general model (mean ± SD of f1-scores in all stages: general model = 0.69 ± 0.02, Y-Y = 0.69 ± 0.03, O-O = 0.67 ± 0.02, *p* < 0.001 for the general model > O-O). The results for each sleep stage showed similar patterns to the overall outcome for all stages. The young group model demonstrated better (in N3: general model = 0.56 ± 0.08, Y-Y = 0.65 ± 0.11, *p* < 0.001 for the general model < Y-Y) or similar performance (in wake: general model = 0.78 ± 0.03, Y-Y = 0.77 ± 0.05; N2: general model = 0.77 ± 0.02, Y-Y = 0.79 ± 0.03; REM: general model = 0.70 ± 0.03, Y-Y = 0.68 ± 0.05) compared with the general model, except for the N1 stage (general model = 0.49 ± 0.03, Y-Y = 0.47 ± 0.04, *p* = 0.001 for the general model > Y-Y). On the other hand, the older group model exhibited significantly lower accuracy in N2 and N3 stages than the general model (in N2: O-O = 0.74 ± 0.03, *p* < 0.001 for the general model > O-O; N3: O-O = 0.30 ± 0.13, *p* < 0.001 for the general model > O-O). There were no statistically significant differences in the other stages (in wake: O-O = 0.77 ± 0.04; N1: O-O = 0.49 ± 0.04; REM: O-O = 0.71 ± 0.04). In addition, with respect to age-specific models, the accuracy when using the validation set from their own age group was relatively higher than that obtained when using the validation set from the other age group (in all stages: Y-O = 0.68 ± 0.02, O-Y = 0.66 ± 0.03, *p* = 0.002 for Y-Y > Y-O, *p* = 0.029 for O-O > O-Y; Supplementary Figure S1).

The patterns of performance differences among the general model and age-specific models identified in the kNN-based models were fairly similar to those identified in the SVM-based models (Figure 2B, middle row). In particular, in terms of overall accuracy for all sleep stages, a statistically significant difference was derived among the models [in all stages: *F*(2,147) = 9.11, *p* < 0.001], and while the younger group model showed no significant difference from the general model, the older group model demonstrated significantly lower accuracy than the general model (in all stages: general model = 0.64 ± 0.02, Y-Y = 0.63 ± 0.03, O-O = 0.62 ± 0.03, *p* < 0.001 for the general model > O-O).

In contrast to the findings in the SVM- and kNN-based models, in MLP-based models, no significant performance differences were identified among the models by age group [in all stages: *F*(2,147) = 2.30, *p* = 0.104; Figure 2B, bottom row]. Compared with the general model, both age-specific models demonstrated no significant difference in the overall accuracy across all stages (in all stages: general model = 0.69 ± 0.02, Y-Y = 0.69 ± 0.03, O-O = 0.68 ± 0.02), even though the younger group model and older group model revealed lower accuracy than the general model for the N1 and N3 stages, respectively (in N1: general model = 0.48 ± 0.04, Y-Y = 0.45 ± 0.05, *p* < 0.001 for general model > Y-Y; N3: general model = 0.56 ± 0.10, O-O = 0.30 ± 0.15, *p* < 0.001 for general model > O-O).

The classification accuracy for sleep stage also demonstrated a significant difference depending on the learning algorithm (in all stages using the general model: *F*(2,147) = 130.57, *p* < 0.001). The SVM- and MLP-based model showed similar levels of accuracy, whereas the kNN-based model yielded significantly lower accuracy than those two models (SVM-based model = 0.69 ± 0.02, kNN-based model = 0.64 ± 0.02, MLP-based model = 0.69 ± 0.02, *p* < 0.001 for the SVM-based model > kNN-based model, *p* < 0.001 for the MLP-based model > kNN-based model). This difference in performance among the models using the different algorithms was consistently identified not only across all stages but also for each sleep stage except for the N3 stage [in wake: *F*(2,147) = 18.17, *p* < 0.001; N1: *F*(2,147) = 89.71, *p* < 0.001; N2: *F*(2,147) = 20.80, *p* < 0.001; N3: *F*(2,147) = 1.37, *p* = 0.257; R: *F*(2,147) = 160.23, *p* < 0.001].

#### 3.1.2. Sleep stage prediction results using the general model

The results of the previous section indicated that the age-specific model may have poorer performance than the general model depending on the age groups. Therefore, we selected the age-integrated general model as a representative model for sleep stage classification and employed it for subsequent work. With respect to the learning algorithm, all three different algorithms, SVM, kNN, and MLP, were used independently.

**Figure 3:**
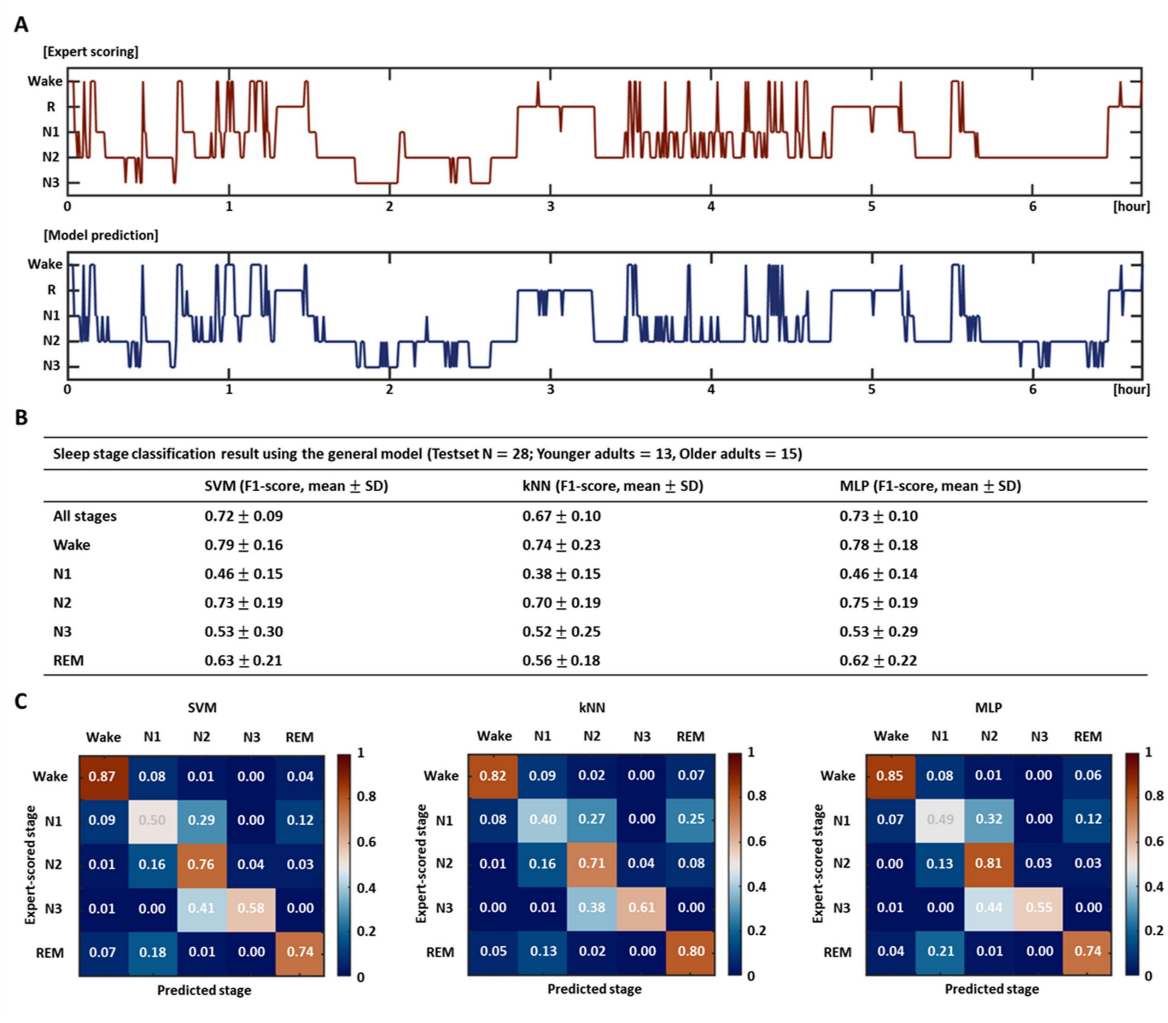
Performance evaluation of sleep stage classification model. **(A)** Test result of a particular subject using the SVM-based general model. The upper red hypnogram represents the expert scored sleep stages, and the lower blue hypnogram indicates the automatically classified stages by the model. **(B)** Sleep stage classification accuracy using the entire test set for each model trained by three different learning algorithms and **(C)** their confusion matrices. The table exhibits the overall accuracy for all stages and the accuracy for each stage. The confusion matrices show the ratios of the predicted stages to the expert-scored stages.

Figure 3A depicts the hypnograms for a single subject (healthy older adult) among a test set of 28 subjects. The upper red graph indicates the expert scored sleep stages, and the lower blue graph shows the automatically classified stages by the SVM-based model. Prediction *via* the model demonstrated an overall similar hypnogram for ~7 h of total sleep and yielded an accuracy (f1-score) of 0.82 in this particular subject. The test results for a total of 28 subjects in each model (age-integrated general model) trained based on the three different learning algorithms are presented in Figures 3B,C. The SVM-, kNN-, MLP-based models revealed average accuracies of 0.72, 0.67, and 0.73, respectively, for all stages. For each stage, they showed high accuracies for the wake and N2 stages but relatively low accuracies for the N1 and N3 stages. Consistent with the model validation results in the previous section, the kNN-based model demonstrated relatively poorer performance than the SVM- and MLP-based models in terms of accuracy at each stage and overall accuracy across all stages; however, in the current test results, the difference in accuracy among the models did not reach statistical significance. The confusion matrices (Figure 3C) visualize the performance of each model at a glance and inform the ratios of the predicted sleep stages to the actual stages (expert-scored sleep stages).

### 3.2. Obstructive sleep apnea screening

#### 3.2.1. Differences in EEG features according to the presence and severity of OSA

**Figure 4:**
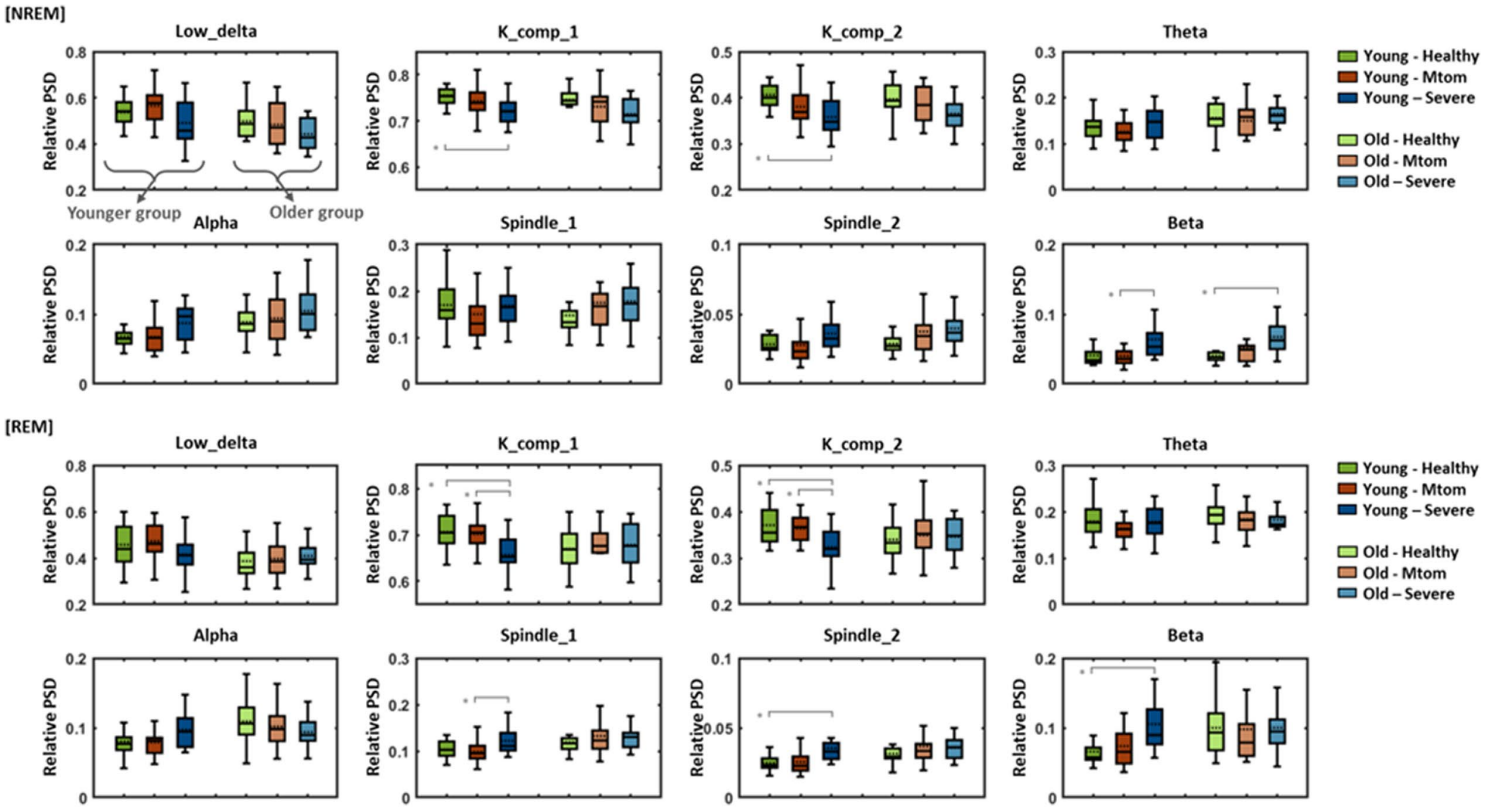
Differences in eight EEG features among healthy subjects, patients with mtom OSA, and patients with severe OSA in the younger and older groups. Results show differences in eight features among the three groups according to OSA severity in REM and NREM sleep stages. Pairs with statistically significant differences are marked by solid gray lines. Asterisk represents a significant difference (*p < 0.01).

Patients with OSA diagnosed based on multiple clinical indices, including respiration-related indices, demonstrated marked differences in the characteristics of sleep EEG as well as in corresponding indices compared with healthy subjects. Such differences in EEG features according to the presence or severity of OSA also differed by age group. Figure 4 shows eight EEG features of healthy subjects, patients with mtom OSA, and patients with severe OSA for the NREM (including N1, N2, and N3) and REM sleep stages in the younger and older groups.

Statistical analysis in the younger group demonstrated significant differences in the K complex, beta-band, and spindle features according to OSA severity [in NREM stages: K_comp_1: *F*(2,53) = 7.61, *p* = 0.001; K_comp_2: *F*(2,53) = 7.67, *p* = 0.001; Beta: *F*(2,53) = 6.10, *p* = 0.004; in REM stages: K_comp_1: *F*(2,53) = 8.90, *p* < 0.001; K_comp_2: *F*(2,53) = 7.31, *p* = 0.002; Spindle_1: *F*(2,53) = 5.10, *p* = 0.009; Spindle_2: *F*(2,53) = 6.21, *p* = 0.004; Beta: *F*(2,53) = 7.01, *p* = 0.002]. In particular, the K complex features (K_comp_1, K_comp_2) exhibited a tendency to decrease toward severe OSA in both the NREM and REM sleep stages, and statistically significant pairs are indicated by gray lines. In contrast, although there was no significant difference between the healthy and mtom OSA groups, the beta-band features showed a tendency to increase toward severe OSA in both sleep stages. In the REM stages, significant differences were elicited in the spindle features (spindle_1 and spindle_2) in addition to the K complex and beta-band features; there was no significant difference between the healthy and mtom OSA groups, but the severe OSA group showed significantly higher values than those two groups.

In contrast to the younger group, differences in EEG characteristics depending on OSA severity were not clearly observed in the older group. In particular, in the NREM stages, K complex and beta-band features represented similar tendencies to those identified in the younger group [only the beta-band features reached statistical significance; *F*(2,53) = 7.57, *p* = 0.001], but no between-group differences (according to OSA severity) were elicited in the REM stages.

#### 3.2.2. Performance analysis of the model

The OSA screening model was built by training the EEG features in the REM/NREM stages (expert-scored stages) and OSA diagnostic results for each subject based on the three learning algorithms. As in the previous section (section 3.1), to examine the age-related effect, the general model and age-specific models were independently generated, and their performance was compared. The models were further evaluated by comparison with models trained on SpO_2_, a respiration-related measure, along with EEG features, to investigate the feasibility of using EEG features.

The average accuracies of the OSA classification (healthy vs. mtom OSA vs. severe OSA) in the general models were 0.46, 0.47, and 0.50 for the SVM, kNN, and MLP algorithms, respectively, when evaluated by performing 10 iterations of five-fold cross-validation; these were far below the average accuracies of the models trained on the EEG features and SpO_2_ (0.64, 0.67, and 0.64 for the SVM, kNN, and MLP algorithms, respectively) (Table 2). However, in the case of the models trained excluding the features of the mtom OSA group, which had intermediate characteristics between the healthy and severe OSA groups, the accuracies considerably increased to 0.74, 0.69, and 0.74 for the SVM, kNN, and MLP algorithms, respectively, even though they did not reach those of the models based on both EEG features and SpO_2_ (0.86, 0.87, and 0.89 for the SVM, kNN, and MLP algorithms, respectively). These patterns were consistently observed in age-specific models as well as the general models.

**Table 2:** Validation of OSA screening model.

Validation results of the OSA screening model (10 iterated five-fold cross validation, N = 111; Healthy = 34, Mtom OSA = 39, Severe OSA = 38)						
	General		Age-specific (Y-Y)		Age-specific (O-O)	
	Model trained with REM/NREM EEG features	Model trained with REM/NREM EEG features + SpO₂ index	Model trained with REM/NREM EEG features	Model trained with REM/NREM EEG features + SpO₂ index	Model trained with REM/NREM EEG features	Model trained with REM/NREM EEG features + SpO₂ index
SVM						
Healthy vs. Mtom OSA vs. Severe OSA	0.46 ± 0.10	0.64 ± 0.09	0.44 ± 0.13	0.67 ± 0.15	0.43 ± 0.13	0.57 ± 0.16
Healthy vs. Severe OSA	0.74 ± 0.12	0.86 ± 0.08	0.74 ± 0.17	0.88 ± 0.08	0.70 ± 0.14	0.83 ± 0.11
kNN						
Healthy vs. Mtom OSA vs. Severe OSA	0.47 ± 0.11	0.67 ± 0.10	0.42 ± 0.16	0.67 ± 0.13	0.42 ± 0.15	0.55 ± 0.15
Healthy vs. Severe OSA	0.69 ± 0.11	0.87 ± 0.07	0.70 ± 0.19	0.88 ± 0.11	0.62 ± 0.15	0.84 ± 0.12
MLP						
Healthy vs. Mtom OSA vs. Severe OSA	0.50 ± 0.11	0.64 ± 0.10	0.48 ± 0.14	0.66 ± 0.15	0.45 ± 0.14	0.55 ± 0.15
Healthy vs. Severe OSA	0.74 ± 0.12	0.89 ± 0.08	0.71 ± 0.16	0.90 ± 0.11	0.72 ± 0.15	0.85 ± 0.12

With respect to the age-related effect, no statistically significant differences were elicited in the performance of the general and age-specific models across all the three learning algorithms. However, in terms of the OSA classification, the age-specific models tended to show slightly lower accuracies than the general model, and the older group model had the lowest accuracy (Table 2). In the healthy vs. severe OSA groups, the younger group model derived a similar accuracy to the general model, but the older group model yielded a relatively lower accuracy than those two models; these characteristics were more pronounced in models trained using the SVM or kNN algorithms.

Finally, regarding the effect of learning algorithms, although MLP-based models demonstrated slightly better performance overall than SVM- and kNN-based models, there was no statistically significant difference.

#### 3.2.3. OSA prediction results using the integrated model combining the sleep stage classification model and OSA screening model

Referring to the results of the analysis in the previous section, the age-integrated general model trained excluding the features of the mtom OSA group was defined as an OSA screening model (healthy vs. OSA), and its performance was evaluated by applying the test set. The test was conducted in a manner that first automatically classified the sleep stages using the model built in the previous section 3.1 and then applied the results to the OSA screening model. The outcomes revealed that the accuracy varied depending on the learning algorithms used in training the sleep stage classification model and OSA screening model (Table 3). In particular, the highest accuracy of 0.73 was derived when the MLP algorithm was applied to both models for training, which indicates that patients with OSA could be screened with the corresponding accuracy level only with sleep EEG characteristics without any respiration-related measures.

**Table 3:** OSA screening results.

OSA screening result using the general model (Testset *N* = 28; Younger adults = 13, Older adults = 15)									
Sleep stage classification model	SVM	SVM	SVM	kNN	kNN	kNN	MLP	MLP	MLP
OSA screening model	SVM	kNN	MLP	SVM	kNN	MLP	SVM	kNN	MLP
	0.65	0.45	0.71	0.58	0.52	0.71	0.65	0.53	0.73

## 4. Discussion

We have proposed an integrated computational framework that can automatically analyze sleep EEG data obtained from each subject, classify sleep stages using machine learning techniques, and determine the risk of OSA based on these findings. The current outcomes have demonstrated the feasibility of AI technologies that can play a beneficial role in clinical applications by enabling automated and systematic analyses.

With respect to automatic sleep stage classification, we constructed a general model that trained on data from all subjects regardless of age and age-specific models that trained only on data from each age group and compared their performance. As a result, the younger group model showed similar accuracy to the general model and even higher accuracies in some stages, although they did not reach statistical significance. In contrast, the older group model exhibited significantly lower accuracies than the general model. In the case of the age-specific models, when the data of subjects belonging to their own age group were used for validation, the accuracy was generally higher than when data belonging to the other age group were used. All these results suggest that there are some differences in sleep EEG characteristics between age groups, as evidenced by the findings of previous studies (Landolt et al., 1996; Campos-Beltrán and Marshall, 2021). In particular, the results imply that the sleep EEG data of younger populations have homogeneous characteristics, given that the younger group model showed similar or higher accuracies despite the smaller training sets than the general model. On the other hand, the performance degradation of the older group model may be interpreted as individual variability increases as aging progresses; thus, the older population has more heterogeneous characteristics.

Previous studies have demonstrated that the older group has larger inter-individual variability than the younger group in terms of not only macro-level sleep architecture (including total sleep time, sleep efficiency, and the ratio of time spent in each sleep stage) but also micro-level architecture that can be identified from the EEG, such as spindle density and REM density (Peters et al., 2014; Mander et al., 2017). Moreover, such characteristics were also observed in the current data set. As a result of examining inter-individual variability based on the Pearson correlation coefficient using the PSD features of each subject’s sleep EEG, a significant difference was found between the two age groups; in general, higher correlation values were derived between individuals in the younger group than in the older group, indicating that the features in the younger group are more homogeneous (Supplementary Figure S2). These group differences were especially pronounced in the wake, REM, and N1 stages. In contrast, in the N3 stage, the older group showed higher correlation values; however, it should be considered that the number of samples for the N3 stage in the older group was significantly smaller than that of the younger group due to the nature of reduced deeper NREM sleep in the older population. The results emphasize that attention should be paid to the bias of individual factors, such as age bias, when training a model using sleep EEG data. Furthermore, future studies are needed to systematically investigate aging-related effects on sleep, especially in terms of changes in brain network characteristics.

The sleep stage scoring accuracy also differed depending on the algorithms used to train the model. Based on the age-integrated general model, the test results for all stages yielded average accuracies of 72, 67, and 73%, when applying the SVM, kNN, and MLP algorithms, respectively, and the SVM- and MLP-based models derived relatively higher performance than the kNN-based model across all stages and for each stage, although not reaching statistical significance. The classification accuracy of sleep stages revealed differences for each stage. While the accuracies for the wake and N2 stages were fairly high, the accuracies for the N1 and N3 stages were relatively low, and these characteristics were consistently observed in the models trained with three different algorithms.

Regarding the N3 stage, the accuracy was somewhat lower than that reported in previous studies (Huang et al., 2014; Zhu et al., 2014; Acharya et al., 2015), and it was often mispredicted as the N2 stage. This outcome may be due to the characteristics of the data set used in this study. To investigate the effects of age and OSA, in the current study, we included more data from patients with OSA as well as older adults compared to the existing studies. In other words, the proportion of older adults and patients with OSA in the entire data set is quite high. The N3 stage represents deep sleep that constitutes approximately 10–20% of total sleep time in healthy people (Kryger et al., 2017), which naturally decreases with aging or as sleep quality deteriorates due to sleep disorders, such as OSA. Therefore, the low accuracy of the model for the N3 stage is likely to be induced by insufficient learning of the features of the corresponding stage, along with increased individual variability with aging, depending on the demographic characteristics of the data set; The model often misclassified N3 as the N2 stage, which was relatively well-trained. This can be improved by acquiring more data sets and training them further.

The low accuracy for the N1 stage and especially the confusion with the N2 stage have been frequently observed in the existing machine learning studies for automatic sleep stage classification (Panossian and Avidan, 2009; Hsu et al., 2013; Supratak et al., 2017; Sors et al., 2018). In fact, both stages belong to shallow sleep and share similar characteristics. According to the AASM manual, the scoring for the N2 stage is based on the occurrence of a K-complex or sleep spindle. However, if the N2 stage has preceded beforehand, the following epoch is also scored as N2 in the absence of arousal or interruption, even if those two features are not observed. In other words, the scoring is performed not only by the features of the sleep EEG but also by the pre-post relationship of the sleep stages. The learning algorithms we applied here, which classify sleep stages by training the PSD features of the sleep EEG for each epoch, have a limitation in that they cannot reflect the scoring considering such a pre-post relationship, thereby exhibiting relatively low performance for the N1 stage. To overcome this issue, it may be an alternative way to use an algorithm that can learn the pre-post data features and use them for stage scoring, such as bidirectional long short-term memory (Zhang et al., 2019; Kuo and Chen, 2020), even at an increased computational cost.

Table 4 presents a summary of the characteristics of several recently published notable EEG-based sleep stage classification models. As expected, models trained on data from healthy subjects with a small age variance demonstrated relatively high overall performance across different learning algorithms compared to other models (Ghasemzadeh et al., 2019). In particular, the model built with data from young, healthy subjects demonstrated the highest accuracy. Direct comparison may be unreasonable given that models can operate at different performance levels depending on the data set, even if the same training approach is applied (Fan et al., 2021); however, the current model exhibited accuracy similar to existing models built on data sets including subjects with sleep disorders and middle-aged adults (75% in Tzimourta et al., 2018, 72% in Tripathy et al., 2020, and 75% with the original unbalanced training approach in Sharma et al., 2021). Recent studies have applied additional processes during model training to improve the classification accuracy of the model. Sharma and colleagues performed unbiased training by equalizing the number of epochs used in each sleep stage learning (i.e., generating a balanced dataset) using over-sampling and under-sampling techniques to resolve the learning imbalance for each sleep stage, which elicited markedly improved performance compared to the training model on the original unbalanced dataset (85% in the balanced dataset and 75% in the original unbalanced dataset; Sharma et al., 2021). Hussain and colleagues used clean preprocessed EEG data by removing signal artifacts (including ocular, muscle, and motion artifacts) *via* independent component analysis and added a feature selection procedure based on further statistical analyses, thereby increasing the accuracy of the model (84–89%; Hussain et al., 2022). The current study aimed to create an EEG-based integrated model for sleep stage scoring and OSA screening and evaluate its feasibility under the condition of minimizing the amount of computation and complexity, considering compatibility with wearable devices and mobile applications, thus, we did not apply additional procedures to improve the model performance. It is expected that models with enhanced performance can be built by applying appropriate additional processes considering the trade-off with computational power, if necessary.

**Table 4:** Summary of existing sleep stage classification models based on conventional machine learning techniques.

Study	Dataset	Subjects N, age, gender	Data channels	Features	Algorithm	Class (the number of epochs for each class)	Accuracy
Tzimourta et al. (2018)	ISRUC-sleep dataset	100 subjects with sleep disorders, 51 ± 16 (mean ± SD), Female: 45 Male: 55	6 channels (F_3_-A_2_, C_3_-A_2_, O1-A_2_, F_4_-A_1_, C4-A_1_, O_2_-A_1_)	Energy for each of 5 subbands ^a^	Naive Bayes	Wake, N1, N2, N3, REM (20,104, 11,104, 27,398, 17,325, 11,256)	56%
					Decision Tree		66%
					kNN		65%
					SVM		67%
					Random Forests		75%
Ghasemzadeh et al. (2019)	ISRUC-sleep dataset	10 healthy subjects, 40 ± 10, Female: 1, Male: 9	1 channel (C_3_-A_2_)	Entropy for each of 11 subbands ^b^ (non-normalized log energy)	SVM	Wake, N1, N2, N3, REM (1702, 1,123, 2,850, 1976, 1,238)	82%
					EBT		81%
					weighted kNN		81%
				Entropy for each of 11 subbands ^b^ (non-normalized Shannon)	SVM		76%
					EBT		79%
					weighted kNN		76%
	Sleep-EDF dataset	8 healthy subjects, 28 ± 5, Female: 4, Male: 4	2 channels (Pz-Oz, Fpz-Cz)	Entropy for each of 11 subbands ^b^ (non-normalized log energy)	SVM	Wake, S1, S2, S3, S4, REM (8,055, 604, 3,621, 672, 627,1,609)	93%
Tripathy et al. (2020)	CAP sleep dataset	25 subjects including 6 healthy subjects and 19 subjects with sleep disorders, 48 ± 14 for female, 64 ± 18 for male, Female: 10, Male: 15	4 channels (P_4_-O_2_, C_4_-A_1_, F_4_-C_4_, C_4_-P_4_)	Dispersion entropy, Bubble entropy for each of 5 subbands ^c^	Hybrid classifier (based on class-specific residuals using sparse representation and distances from nearest neighbors)	Wake, S1, S2, S3, S4, REM (2,613, 1,537, 4,955, 2,707, 2,601, 2,947)	72%
Sharma et al. (2021)	CAP sleep dataset	80 subjects including 6 healthy subjects and 74 subjects with sleep disorders, 48 ± 20, Female: 32, Male: 48	2 channels (F_4_-C_4_, C_4_-A_1_)	Norm features ($l_{1},l_{1}$, $l_{\infty }$) and their statistics from 6 subbands signals (obtained from wavelet decomposition)	Decision tree	Wake, S1, S2, S3, S4, REM for original unbalanced epochs: (15,841, 3,519, 28,628, 8,804, 10,188, 13,687) for balanced epochs: (13,407, 13,407, 13,407, 13,407, 13,407, 13,407)	With EBT, for unbalanced training: 75%, for balanced training: 85%
					Logistic regression		
					Naive Bayes		
					SVM		
					kNN		
					EBT		
Hussain et al. (2022)	HMC sleep staging dataset	100 subjects with sleep disorders, 54 ± 15 Female: 66, Male: 88	3 channels (F_4_, C_4_, O_2_)	Mean power, median frequency, mean frequency, spectral edge, peak frequency for each of 5 subbands^d^, delta-alpha power ratio, delta-theta power ratio, slow-fast wave power ratio	C5.0 decision tree	Wake, S1, S2, S3, S4, REM	87%
					MLP		89%
					CHAID		84%

a Subbands, 0–4, 4–8, 8–13, 13–30, and 30–60 Hz; ^b^subbands, 0.4–4, 4–8, 8–10, 10–13, 13–18, 18–25, 25–30, 30–36, 36–41, 41–46, and 46–50 Hz; ^c^subbands, 0–4, 4–8, 8–13, 13–30, and 30–75 Hz; ^d^subbands, 0.5–4, 4–8, 8–13, 13–30, and 30–44 Hz; ISRUC, Institute of Systems and Robotics, University of Coimbra; CAP, Cyclic Alternating Pattern; HMC, Haaglanden Medisch Centrum; kNN, k-Nearest Neighbors; SVM, Support Vector Machine; EBT, Ensemble Bagged Trees; MLP, MultiLayer Perceptron; CHAID, Chi-Squared Automatic Interaction Detector.

Characteristics of sleep EEG at each NREM and REM stage demonstrated significant differences between healthy subjects and patients with OSA, consistent with previous findings (Kang et al., 2021). Those differences were more pronounced in the younger group than in the older group. The younger group demonstrated significant differences in K complex and beta band features across NREM and REM stages according to the presence or severity of OSA (healthy vs. mtom OSA vs. severe OSA groups). The group showed additional significant differences in spindle features in the REM stage. The older group exhibited relatively similar patterns to the younger group in the NREM stage, but a significant difference was derived only for the beta band feature, and no noticeable differences (among the three groups) were found in the REM stage. Accordingly, the OSA classification model trained on EEG features in NREM and REM stages represented generally lower accuracy in the older age-specific model than the younger age-specific or general model. Concerning the learning algorithm used for model training, the MLP-based model yielded higher performance than the SVM- and kNN-based models, but the difference was not statistically significant.

The MLP-based OSA screening model trained excluding the features of the mtom OSA group, which has intermediate features that are relatively indistinguishable compared to the healthy or severe OSA groups, exhibited 73% of performance when applying the scoring results derived from the automatic sleep stage classification model. These results are critically meaningful in that the model was able to distinguish patients with OSA (mtom and severe OSA) from healthy subjects, using only the characteristics of the sleep EEG without respiration-related measures. The results further suggest the need for systematic studies for OSA disease in terms of brain networks, including whether the altered brain network properties cause respiration-related problems during sleep or vice versa. Regarding OSA screening, another feature that needs to be carefully investigated in sleep EEG is arousal. In fact, one of the main characteristics of patients with OSA is frequent arousals from sleep, which are accompanied by a sudden increase in EEG frequency (Altevogt and Colten, 2006; Yue et al., 2009). In the dataset used in the current study, a significant difference was observed in the number of arousals during sleep among the healthy, mtom, and severe OSA groups. Furthermore, the performance of the OSA screening improved when the model was trained by adding the number of arousals in addition to NREM/REM EEG features (Supplementary Figure S3). Although the current study did not cover the contents related to the arousal index in-depth, given that the recent findings demonstrated that gamma power in EEG arousal differs according to the severity of the respiratory event and sleep stages (Pitkänen et al., 2021), future studies may need to closely examine such characteristics and incorporate them into building the models as needed.

Although the current results showed the feasibility that the OSA screening could be achieved to some extent with only EEG characteristics, the accuracy still did not reach that of the model trained by adding the respiratory-related index, SpO_2_, as features. This is in line with the results of a recent paper demonstrating that a deep learning-based model trained on pulse oximetry measures is effective in scoring sleep stages and estimating AHI (Huttunen et al., 2022), and suggests that SpO_2_ monitoring may be important in increasing efficacy for the screening of, at least, OSA, among several other sleep disorders. Given that SpO_2_ could be obtained through a relatively simple setup, such as a finger pulse oximeter, current state-of-the-art wearable devices, which are being developed for individual sleep quality evaluation (Koushik et al., 2019; Liao et al., 2020) may be expanded into integrated systems that include detecting SpO_2_ levels as well as the EEG. Such systems would be particularly useful in enabling individuals to analyze their sleep patterns and assess their risk of sleep disorders, even at home.

In the current study, an integrated model was constructed that performs sleep stage classification and OSA screening based on sleep EEG, and its feasibility was verified. However, there are still several limitations, and further studies are needed. To investigate age-related effects, we divided the subjects into two age groups, built a model for each group or the entire group, and elicited meaningful results by comparing their performance, but due to the limitations of the data set, data from older adults aged 65 years or older were not included. Therefore, it is required to expand the model based on sleep EEG obtained from older adults and to further verify the current outcomes. In addition, due to the nature of OSA, where the rate of diagnosis is 3.3 times higher in men than women (Bixler et al., 2001), there was a gender bias in the current data set (men: 107, women: 32). Moreover, there was a difference in the gender ratio between the two age groups. Such a gender bias may have affected the current results, as previous studies have demonstrated significant differences in sleep EEG characteristics depending on gender groups (e.g., higher PSDs in women, especially in delta, theta, low alpha, and high spindle frequency bands; Carrier et al., 2001; Simor et al., 2013). Thus, it is necessary to systematically analyze the effects of gender by using data sets without gender bias or by comparing models built according to gender.

As mentioned earlier, we here used relatively less complex and low-computational training approaches to determine the feasibility of the integrated model. The accuracy of the model could be further improved by removing signal artifacts in the sleep EEG, selecting only clean epochs and applying them to the model training, or employing a more complex architecture, such as recurrent neural networks that can reflect time-varying dynamic features. In addition, regarding the features used for model training, frequency-domain features, which can directly quantify important patterns in sleep stage scoring with simple computations, were used in the current study. However, given that previous studies used various types of features including time-domain and nonlinear features in addition to frequency-domain features, and demonstrated quite good model performance (Aboalayon et al., 2016), further studies are needed to investigate the effects of features by performing comparative analysis on diverse features. Applying dimensionality reduction or feature selection algorithms could further increase the model efficiency.

Many studies are currently underway to provide individual assistance for health management through wearable devices and relevant mobile applications (Koushik et al., 2019; Liao et al., 2020). The current outcomes suggest that AI-based computational studies, combined with such innovative technologies, can not only evaluate sleep status in an individual manner but also promote early intervention by informing the risk of sleep disorders such as OSA. Those studies could ultimately contribute to personalized medicine.

## Data availability statement

The original contributions presented in the study are included in the article/Supplementary material, further inquiries can be directed to the corresponding author.

## Ethics statement

The studies involving human participants were reviewed and approved by Institutional Review Board of Ewha Womans University Mokdong Hospital (approval No. EUMC 2018–10-008). Owing to the retrospective and anonymized nature of this standard PSG database, the review board waived the need to obtain the patients’ informed consent.

## Author contributions

CK, SA, and HL contributed to conception and design of the study. CK and SA contributed to construction of computational framework and interpretation of the data, and wrote the manuscript. HK, MD, HL, and SH contributed to data acquisition and data curation. HL contributed to data resources, interpretation of the data, funding acquisition, and substantively revised the article. All authors contributed to the manuscript and approved the submitted version.

## Funding

This work was supported by grants from Institute of Information and communications Technology Planning and Evaluation (IITP) funded by the Korea government (MSIT) [No. RS-2022-00155966, Artificial Intelligence Convergence Innovation Human Resources Development (Ewha Womans University)], the Basic Science Research Program and Convergence Technology R&D Program for Human Augmentation (NRF-2019M3C1B8090803 and 2020R1A2C2013216), and by the BK21 FOUR (Fostering Outstanding Universities for Research) though the National Research Foundation of Korea (NRF) by the Korean government to HL. This work was also supported by the Basic Science Research Program through the NRF funded by the Ministry of Education (NRF-2020R1I1A1A01073605) to SA, and the Korea Health Technology R&D Project through the Korea Health Industry Development Institute funded by the Korean Ministry of Health & Welfare (HI19C1065) to HK.

## Conflict of interest

The authors declare that the research was conducted in the absence of any commercial or financial relationships that could be construed as a potential conflict of interest.

## Publisher’s note

All claims expressed in this article are solely those of the authors and do not necessarily represent those of their affiliated organizations, or those of the publisher, the editors and the reviewers. Any product that may be evaluated in this article, or claim that may be made by its manufacturer, is not guaranteed or endorsed by the publisher.
