# Supplementary material for: Age-integrated artificial intelligence framework for sleep stage classification and obstructive sleep apnea screening
Source: Front Neurosci. 2023 Jun 14;17:1059186. doi: 10.3389/fnins.2023.1059186 (PMC10300414; doi:10.3389/fnins.2023.1059186)
Supplement: Supplementary file 1 [file Data_Sheet_1.PDF]

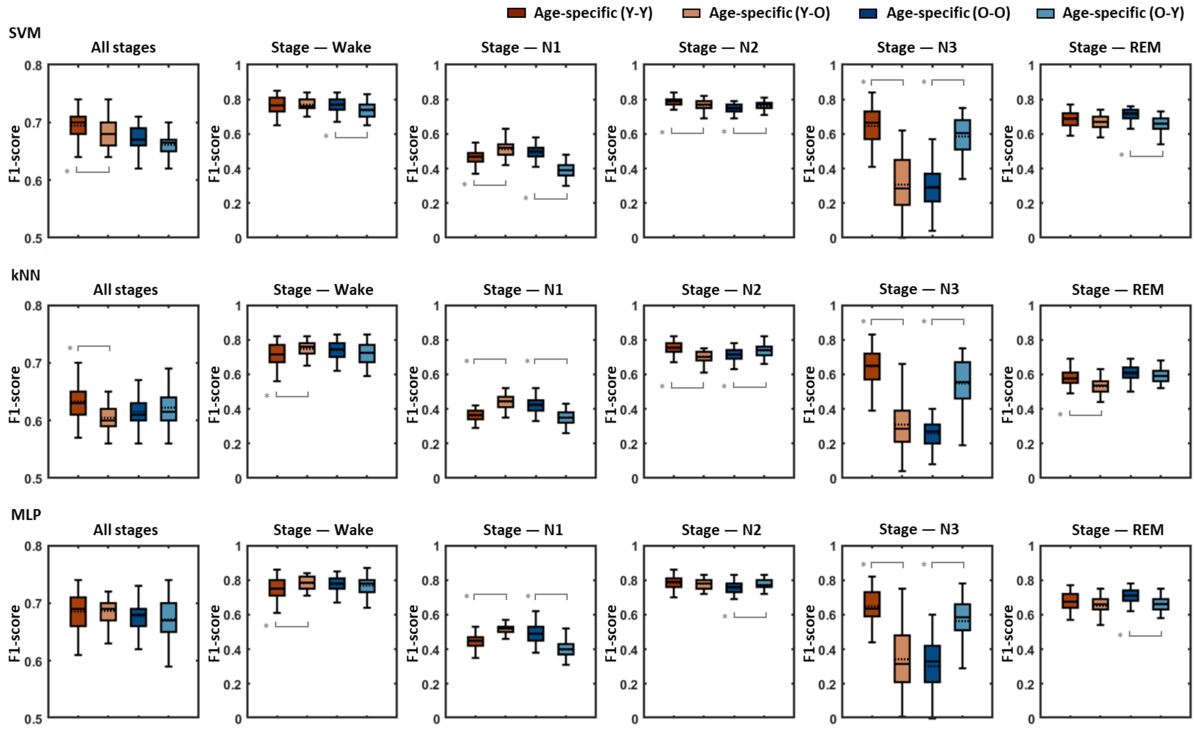

Supplementary Fig S1. Validation results for age-specific models. The figures represent the validation results performed using data belonging to their own age group (Y-Y, O-O) as well as data from the other age group (Y-O, O-Y), in age-specific models built on three different learning algorithms. Pairs with statistically significant differences in classification accuracy are indicated by solid gray lines. An asterisk represents a significant difference (* p < 0.01).

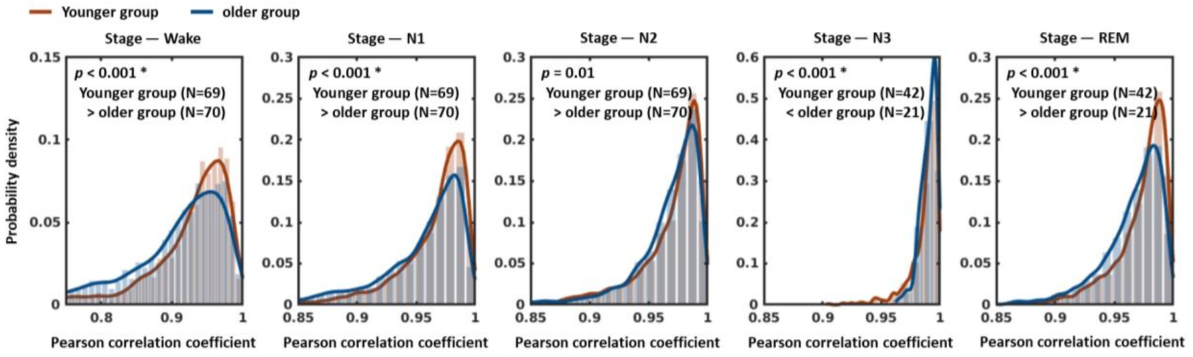

Supplementary Fig S2. Inter-individual variability of sleep EEG features in the younger and older groups. The figure shows distributions of correlation coefficients between individual EEG features in each sleep stage. The red and blue lines represent the results in the younger and older groups, respectively, and asterisks indicate statistically significant differences (*$p < 0.01$).

A

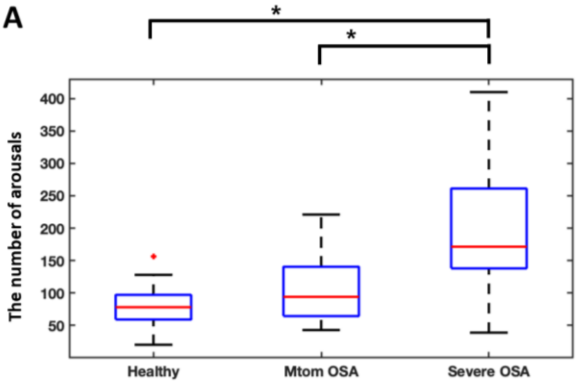

B

Validation results of the OSA screening model (10 iterated five-fold cross validation, N = 111; Healthy = 34, Mtom OSA = 39, Severe OSA = 38)						
General model	SVM		kNN		MLP	
	Healthy vs. Mtom OSA vs. Severe OSA	Healthy vs. Severe OSA	Healthy vs. Mtom OSA vs. Severe OSA	Healthy vs. Severe OSA	Healthy vs. Mtom OSA vs. Severe OSA	Healthy vs. Severe OSA
Model trained with REM/NREM EEG features	0.46 ± 0.10	0.74 ± 0.12	0.47 ± 0.11	0.69 ± 0.11	0.50 ± 0.11	0.74 ± 0.12
Model trained with REM/NREM EEG features + the number of arousals	0.47 ± 0.09	0.84 ± 0.10	0.45 ± 0.08	0.83 ± 0.11	0.51 ± 0.09	0.81 ± 0.10

Supplementary Fig S3. Effect of the number of EEG arousals on OSA screening. (A) The number of EEG arousals in healthy, mtom OSA, and severe OSA groups. Each boxplot shows the median, quartile, minimum and maximum values for the number of arousals in each group. Red crosses indicate outliers and black asterisks represent group pairs that revealed statistically significant differences (*$p < 0.01$). (B) Comparison of OSA screening accuracy between a model trained with only EEG features and a model trained with both EEG features and the number of arousals. The table presents the results of applying the three different algorithms, SVM, kNN and MLP.
